# Treatment of preterm brain injury via gut‐microbiota–metabolite–brain axis

**DOI:** 10.1111/cns.14556

**Published:** 2023-12-18

**Authors:** Ling Li, Tianjing Liu, Yongyan Shi

**Affiliations:** ^1^ Department of Pediatrics Shengjing Hospital of China Medical University Shenyang China

**Keywords:** dietary intervention, drugs, fecal microbiota transplantation, gut–brain axis, preterm infant, probiotics

## Abstract

**Background:**

Brain injury in preterm infants potentially disrupts critical structural and functional connective networks in the brain. It is a major cause of neurological sequelae and developmental deficits in preterm infants. Interesting findings suggest that the gut microbiota (GM) and their metabolites contribute to the programming of the central nervous system (CNS) during developmental stages and may exert structural and functional effects throughout the lifespan.

**Aim:**

To summarize the existing knowledge of the potential mechanisms related to immune, endocrine, neural, and blood–brain barrier (BBB) mediated by GM and its metabolites in neural development and function.

**Methods:**

We review the recent literature and included 150 articles to summarize the mechanisms through which GM and their metabolites work on the nervous system. Potential health benefits and challenges of relevant treatments are also discussed.

**Results:**

This review discusses the direct and indirect ways through which the GM may act on the nervous system. Treatment of preterm brain injury with GM or related derivatives, including probiotics, prebiotics, synbiotics, dietary interventions, and fecal transplants are also included.

**Conclusion:**

This review summarizes mechanisms underlying microbiota‐gut‐brain axis and novel therapeutic opportunities for neurological sequelae in preterm infants. Optimizing the initial colonization and microbiota development in preterm infants may represent a novel therapy to promote brain development and reduce long‐term sequelae.

## Graphical abstract

Treatment of preterm brain injury via gut‐microbiota‐brain axis.

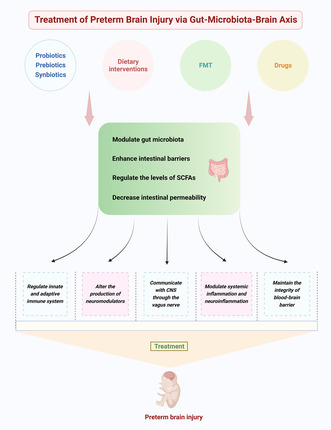

## 1 INTRODUCTION

With the global incidence of approximately 15 million per year, prematurity is associated with a high rate of brain injury.^1^ The premature infants feature underdeveloped nervous systems owing to their early separation from their mothers, and the infants may have to experience prenatal infections, hypoxia–ischemia, and another postnatal injury that potentially increase their risk of brain injury.^2^ Preterm brain injury may result in periventricular leukomalacia, cerebral palsy, attention deficit, hyperactivity disorder, autism, and other behavioral and cognitive deficits.^3^, ^4^ Currently, there is no established to prevent or mitigate the brain injury in preterm infants. Previous studies have highlighted promising neuroprotective treatments, such as therapeutic hypothermia, prenatal and postnatal corticosteroid administration, human amniotic epithelial cell therapy, supplementation with umbilical cord blood, vitamin D, melatonin, erythropoietin, and certain histone deacetylase inhibitors.^5^

Increasing evidence suggests that changes in gut microbiota (GM) coincide with the early development of the nervous system. Initial colonization and developmental optimization of the GM may influence brain development and improve neurological outcomes.^6^ This suggests that early intervention may significantly affect microbiota maturation and improve neurodevelopmental outcomes in preterm infants.^7^ An integrated longitudinal profiling study of 60 preterm infants substantiated the crucial role of the gut microbiota‐immune‐brain axis in brain injury in preterm infants.^8^ Early dysbiosis and changes in associated metabolites can lead to hippocampal dysfunction and behavioral impairment in mice with antibiotic‐modified gut microecological dysregulation, highlighting the potential of microbiome‐mediated therapies for psychiatric disorders.^9^ Gut microbiome‐mediated interventions represent potential therapeutic roles in the neuroprotective strategy of preterm infants.

This review discusses the synchronous process of neurodevelopment and GM maturation in newborns, as well as the potential mechanisms of communication between GM, metabolites, and the immature brain. Therapeutic vehicles such as probiotics, prebiotics, synbiotics, dietary interventions, fecal microbiota transplantation (FMT), and natural drug‐related gut–brain axis pathway interventions are summarized, aiming to identify promising therapeutic measures for neurocognitive deficits and neurological disorders associated with prematurity.

## 2 OVERVIEW OF PRETERM BRAIN INJURY

Brain injury is a common complication of preterm infants, including Intraventricular hemorrhage, periventricular leukomalacia, and diffuse white matter injury (WMI).^10^ In very low birth weight preterm infants, the germinal stroma blood vessels are immature and the tissue blood vessels are poorly supported, making the lateral ventricle particularly susceptible to intraventricular hemorrhage, white matter coagulation, and necrosis.^11^, ^12^ As the most common form of preterm brain injury, periventricular WMI or periventricular leukomalacia often occurs in infants between 24 and 32 weeks of gestational age, which may be accompanied by reduced cortical volume, thalamus, and basal ganglia volume.^13^ Necrotic lesions can develop into multiple cystic lesions within a few weeks, which is the most severe injury leading to cerebral palsy.^14^ Diffuse WMI is associated with impaired cognitive, sensory, and psychological functions, and is increasingly recognized as a risk factor for autism spectrum disorders, attention deficit hyperactivity disorder, social difficulties, anxiety, behavioral disorders, and other psychological disorders.^15^, ^16^

Prenatal or postnatal factors (such as intrauterine infection and/or inflammation, fetal immune response syndrome, and hypoxia–ischemia) may affect the pathophysiology of preterm brain injury and is related to adverse neurological outcomes.^17^ These factors may trigger oxidative stress reaction to produce a large number of oxygen free radicals (FRs). Because the brain of preterm infants cannot activate antioxidant defense, it is particularly vulnerable to OS damage and sensitive to FRs, which causes neuronal degeneration, edema, death, and can lead to cerebral palsy, cognitive, behavioral, and attention deficits.^18^, ^19^ FRs have also been shown to disrupt the maturation and differentiation of pre‐oligodendrocytes into mature oligodendrocytes, thus leading to WMI, which is the dominant mode of brain injury in preschoolers.^20^

Interestingly, it has been demonstrated that the microbiome and its metabolites can regulate oxidative stress‐induced damage, affecting the differentiation and myelination of precursor oligodendrocytes.^21^ Brittany Needham and his colleagues observed that 4‐ethylphenol sulfate, an intestinal‐derived metabolite, can enter the brain of mice with neurodevelopmental disorders, and promote anxiety‐like behavior by affecting oligodendrocyte function and neuronal myelin sheath pattern in the brain.^22^ Ciara E Keogh and colleagues studied ABX‐induced intestinal disorders in mice and observed that antibiotic treatment can lead to up‐regulation of myelin‐related genes in the prefrontal cortex, mature oligodendrocyte precursor cells, increase in myelin production, and impairment to hippocampal neurogenesis. Supplementation of tributyl glyceride, a metabolite of intestinal bacteria, can effectively alleviate behavioral defects, intestinal pathology, and myelin disorder in mice.^23^ Another mouse study found that trimethylamine N‐Oxide, a metabolite of GM, can cross the BBB through NLR family pyrin domain‐containing 3 (NLRP3) inflammasome signaling and mitochondrial dysfunction, promoting oligodendrocyte pyroptosis and demyelination.^24^ In the future, the specific role of GM in myelination may be further investigated by using myelin‐specific knockout mouse models.

## 3 MAJOR PROCESSES IN NEURODEVELOPMENT COINCIDE WITH CHANGES IN THE NEONATAL GUT MICROBIOME

Neurodevelopment can be roughly divided into neural induction, neural pattern formation, neurogenesis, and axon growth.^25^ Recent studies have found that basic neurodevelopmental processes are synchronized with the maturation of GM in newborns. Oliphant et al. investigated the relationship between head circumference growth and the establishment of GM from the first week of life by collecting fecal samples from 58 preterm infants, finding that poor head circumference growth was associated with low abundance of *Bacteroidetes* and *Uncaria*.^26^ Another prospective observational study explored the relationship between the dynamic sequence of *Bifidobacterium* in extremely low birth weight infants during the first month of life and their neurodevelopment at 24 months of corrective age. *Bifidobacteria* in preterm infants with normal and damaged neurodevelopment followed different time trajectories and had different component rearrangements.^27^ This possibly indicates that the developmental trajectory of the nervous system might somehow be shaped by the maturation of GM. Moreover, GM participates in perinatal brain development, including neurogenesis, microglial maturation, BBB development, and myelination.^28^, ^29^, ^30^, ^31^ In the future, it is possible to interfere with neurodevelopmental trajectories by early regulation of GM and their metabolites.

## 4 POTENTIAL MECHANISMS BY WHICH GUT MICROBIOTA AFFECTS NEURODEVELOPMENT

**FIGURE 1:**
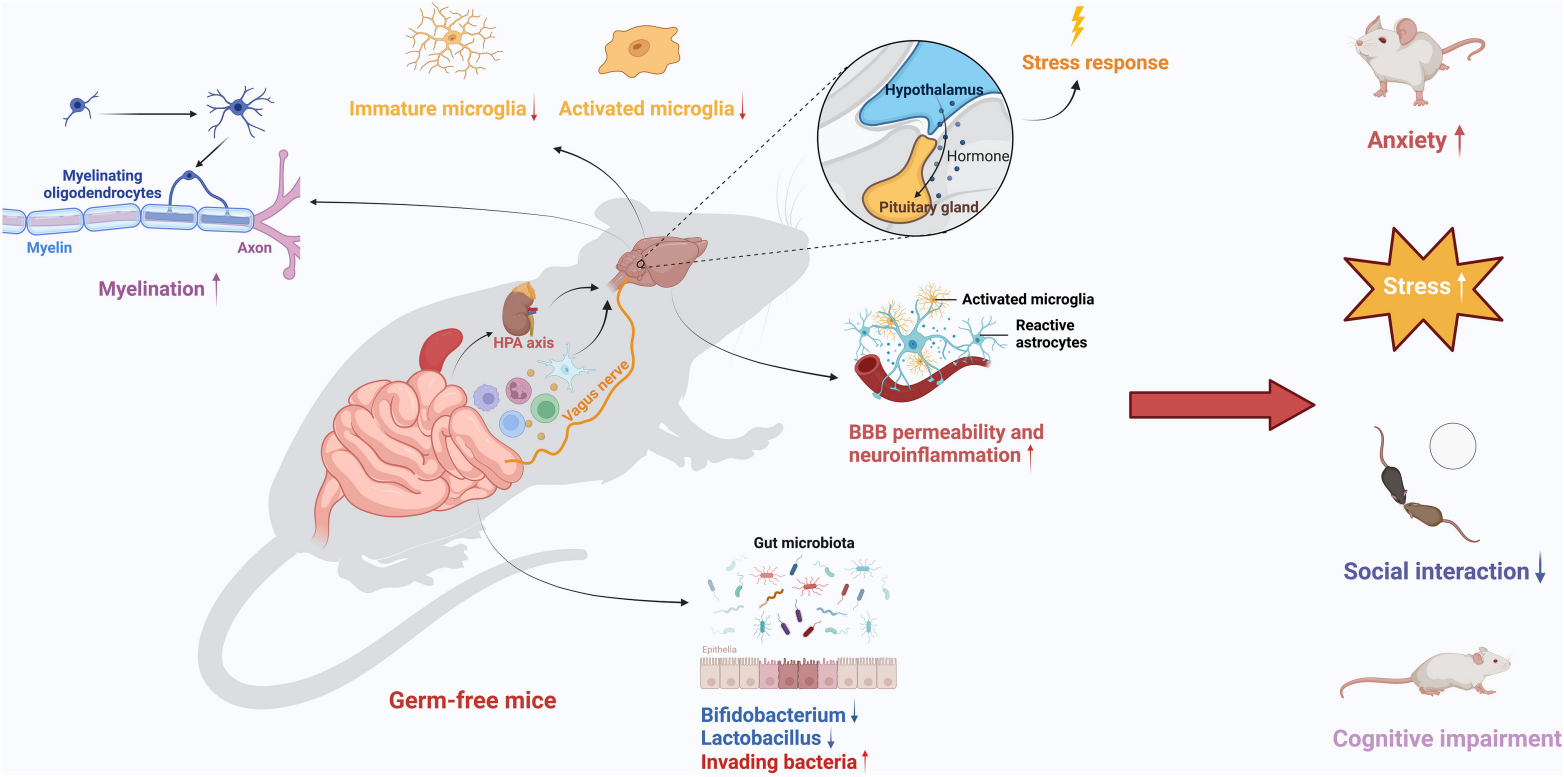
Neurophysiological and behavioral changes were induced by intestinal microbial defects in germ‐free mice. Germ‐free mice have intestinal microbial defects and are prone to abnormal intestinal microbial colonization after birth. Intestinal microbiota may regulate brain function and development, such as myelination, microglia maturation, HPA axis development, HPA axis stress response, and the development and maintenance of BBB integrity through immune signal transduction, endocrine, and neural pathways. Therefore, germ‐free mice were prone to stress, reduced social interaction, and cognitive deficits. *HPA* hypothalamic–pituitary–adrenal; *BBB* blood–brain barrier (Figure created with Biorender.com).

Early in life, the GM maturation coincides with crucial neurodevelopmental period, during which time the neural circuits are highly plastic and vulnerable.^32^ Experiments in germ‐free mice have confirmed the importance of the GM in many fundamental processes in the brain, including BBB permeability, brain volume, neural circuits, myelination, and microglial alterations^33^, ^34^, ^35^ (Figure 1). These processes are critical in shaping animal behavior and neurodevelopment and may involve the role of GM and their metabolites.^36^

### 4.1 Immune system

Innate immune cells can recognize pathogen‐associated molecular patterns through pattern recognition receptors and rapidly induce non‐specific inflammatory responses.^37^ GM have an important influence on brain development by regulating the production of components of innate immune response, macrophages, microglia, and cytokines.^38^ Toll‐like receptor 4 (TLR4) has been shown to mediate microbiome‐brain communication.^39^ Huiling Wei and colleagues observed in mice that the dietary microbial metabolite butyrate inhibits microglia‐mediated neuroinflammation and regulates the microbiota–gut–brain axis (MGBA) by binding to GPR109A, upregulating PPAR‐γ expression, and downregulating TLR4 and Nuclear factor Κb (NF‐κB) activation, improving memory and cognitive function.^40^ In addition, ProBiotic‐4 inhibits bacterial‐related TLR4 and NF‐κB signaling pathway shows improvement in memory impairment, synaptic and brain neuron damage, and microglial activation.^41^

Microglia are innate immune cells of the brain that fine‐tune neurogenesis and synaptogenesis by secreting pro‐inflammatory cytokines such as tumor necrosis factor‐alpha (TNF‐α), IFN‐α, and interleukin‐1β (IL‐1β). Meanwhile, microglia are important neuroimmune components of the MGBA.^42^, ^43^ Studies using germ‐free mice have shown changes in cell size and proportion in microglia, as well as their abnormal development and function.^44^ Dysbiosis can significantly affect the cell‐specific transcriptome, especially in microglia and their subtypes.^45^ Moreover, changes in GM caused by diet, exercise, and antibiotics can act on microglia and then affect the recombination of neural networks,^46^ and alter the neurophysiology of adolescence, including the expression of myelin‐related genes in the prefrontal cortex and the morphology of microglia in the basolateral amygdala.^47^ In another CLP‐induced septicemia study in rats, the diversity and abundance of *Bacteroides distasonis, Lactobacillus salivarius*, and *Clostridium cluster* decreased, the activation of microglia increased, and the inflammatory factors in hippocampus and cortex increased.^48^ Neuroinflammatory responses and microglia synaptic damage may induce neuropsychiatric disorders related to rodent behavioral phenotype.^49^

Gut microbiota is widely and comprehensively involved in the shaping and function of the adaptive immune system.^50^ GM not only induces the differentiation and maturation of antigen‐specific T‐ and B cells by producing specific antigens and metabolites, but also interacts with lymphocytes in the intestinal mucosal immune system to play a part in CNS diseases. By analyzing the microbiome characteristics of stress‐sensitive mice, it was found that *Lactobacillus* (involved in T‐cell differentiation to protect host immune system) decreased, and the differentiation of IL‐17‐producing colon γ δ T cells (γ δ 17 T cells) increased. Intestine γδ 17 T cells can migrate to the meninges and promote inflammation, thereby promoting stress‐induced social avoidance behavior in mice.^51^, ^52^, ^53^ In addition, peripheral‐induced autoreactive myelin‐specific T cells can be activated by *Lactobacillus reuteri* through molecular simulation after migrating to the gut, and then, highly pathogenic Th17 cells may enter the CNS to induce demyelination response.^54^

### 4.2 Neuroendocrine system

The GM can produce various metabolites, including short‐chain fatty acids (SCFAs), bile acid metabolites, neurotransmitters (such as GABA, 5‐hydroxytryptamine (5‐HT)), tryptophan, catecholamines, and its derivatives, which can affect the neural and endocrine pathways in neurodevelopment. Bile acid receptors, including G protein‐coupled bile acid receptor 5 and farnesol X receptor, are expressed in the brain, mainly in the cerebral cortex, hippocampus, and hypothalamus. Bile acids play a role through the activation of nuclear hormone farnesol X receptor) and G protein‐coupled bile acid receptor 5.^55^ GM is an important regulator of bile acid metabolism. The diversity of bile acid is decreased in germ‐free and antibiotic‐treated rats, and bile acid metabolites can activate receptors and act as signal molecules in the host, which can affect HPA axis and play a role in the brain.^56^ Intestinal *Lactobacillus* improved synaptogenesis and maintained glial homeostasis by balancing the metabolism of tryptophan‐derived neurotransmitters, increasing the levels of 5‐HT in brain and colon, and increasing the levels of BDNF and gamma‐aminobutyric acid receptors in the hippocampus and amygdala.^57^, ^58^ In addition, SCFAs can stimulate the synthesis and secretion of 5‐HT in intestine, which plays a role in regulating motility, neurodevelopment and differentiation, and emotion.^59^ Moreover, colonization of *Lactobacillus* or supplementation of SCFAs can increase the level of GABA in the hippocampus of mice, regulate the level of specific neurotransmitters in hippocampus of brain, and play a role in enhancing memory.^60^

The ecological perturbations to the *Bifidobacteria, Proteobacteria, Lactobacillus, and Staphylococcus* in early life may alter the signaling associated with developmental programs during the critical developmental trajectory of the stress axis.^61^ In infants and preterm infants, who are vulnerable to gut microenvironment interventions, dysbiosis may interfere with hypothalamic–pituitary–adrenal (HPA) axis‐related signaling, inducing cognitive or behavioral changes.^62^ The experiments in germ‐free mice showed elevated corticosteroid levels and stress‐anxious behaviors under mild stress. These behaviors were normalized when the mice were colonized with *Bifidobacterium longum* subsp.^63^

In response to stress, the HPA axis regulates cortisol and catecholamine secretion, directly affecting the intestinal permeability and barrier function, which interferes with the intestinal microbiota composition.^64^ The experiments in ERβ‐deficient mice have shown that dextran sodium sulfate‐induced colitis causes over‐activation of the HPA axis, which might be associated with anxiety‐like behaviors.^65^ In addition, chronic unpredictable mild stress can reduce the catecholamine synthesis ability of the adrenal gland, disrupt the tryptophan and serotonin metabolic pathways in the hippocampus, prefrontal cortex, and colon, and alter the composition of the gut microbiota, inducing anxiety‐like behavior.^66^ Some specific probiotics may inhibit excessive stress responses in social interactions and improve emotional and anxiety symptoms in response to chronic stress by restraining corticosterone and catecholamine production.^67^ Probiotics not only improves depressive behavior by increasing glucocorticoid receptor expression and decreasing adrenocorticotropin‐releasing factor expression, adrenocorticotropic hormone, and corticosterone levels in the blood,^68^ but also attenuates the homeostasis disruption caused by stress, regulates cortical brain‐derived neurotrophic factor (BDNF) expression, and inhibits over‐response to stress in the hippocampus and HPA axis.^69^

### 4.3 Vagus nervous system

The vagus nerve is considered the most direct pathway for microbiota signals to reach the brain. It receives signals from gut microbial metabolites and neurotoxic agents as well as directly synapses with enteroendocrine cells to transmit sensory signals to the nucleus accumbens, projecting many behavior‐related areas of the brain.^70^ The neuronal recordings and imaging in mice revealed that compounds from intestinal bacteria emit signals through the vagus nerve that transmit intestinal osmotic pressure fluctuations to the brain to modulate thirst and drinking behavior.^71^ Besides, the impairment on the gut and neurodevelopment may stem from abnormal or pathological vagal connections.^72^ Therefore, normalizing the function of the vagus nerve may restore neuronal cell homeostasis and optimize brain development, providing a promising strategy for preventing prematurity‐associated neurological disorders.

### 4.4 The blood–brain barrier

Two main barriers exist between the microbiota and brain: the gastrointestinal barrier and BBB. The permeability of these barriers is closely related to the transport of microbiota‐derived metabolites and neurotransmitters in the MGBA.^73^ Animal experiments revealed that increases ratio of the *Firmicutes* to *Bacteroidetes* and associated metabolite changes activated NLRP3 inflammatory vesicles in the colon and brain, disrupting the functional integrity of the BBB, stimulating neuroinflammation, and leading to brain cell apoptosis.^74^, ^75^ The expression of occludin and claudin‐5 decreased in the hippocampus and prefrontal cortex after long‐term high‐fat diet intake, leading to impaired brain function and neurobehavioral changes involving mood, social skills, learning, and memory.^76^ Correspondingly, maternal GM is involved in regulating the BBB of the fetus in utero by upregulating the expression of tight proteins.^77^

The deficits in BBB development in early life impair subsequent spatial learning. Maternal supplementation with the *Lactobacillus reuteri* and their metabolite short‐chain fatty acids (SCFAs) may rescue the cognitive impairment associated with fetal BBB dysplasia and dysfunction.^78^ Studies in germ‐free mice have shown that gut‐derived SCFAs may be a potential epigenetic regulator of learning, memory formation, and storage.^79^, ^80^ Researchers found that BBB permeability in germ‐free mice increased in utero and remained unchanged after birth till adulthood. Supplementation with SCFA‐producing *Clostridium tyrobutyricum* restored the homeostasis in the gut and brain.^81^ It is because SCFAs may maintain intestinal barrier and BBB integrity by regulating tight junction proteins.^82^ SCFAs also work with microglia and mitochondria to regulate BBB function and neuroinflammation.^83^ Although microbial‐derived SCFAs are beneficial to the host by promoting intestinal health and BBB integrity, excessive SCFAs have been demonstrated to be harmful or even toxic.^84^

It seems a promising treatment for preterm infants with brain injury by targeting at factors working in the MGBA. However, as far as we know, the potential mechanistic pathways by which GM affects neurodevelopment are not fully verified in preterm brain injury, and more clinical studies are needed to elucidate its mechanism.

## 5 TREATMENT OF BRAIN INJURY IN PRETERM INFANTS MEDIATED THROUGH THE GUT–BRAIN AXIS

**FIGURE 2:**
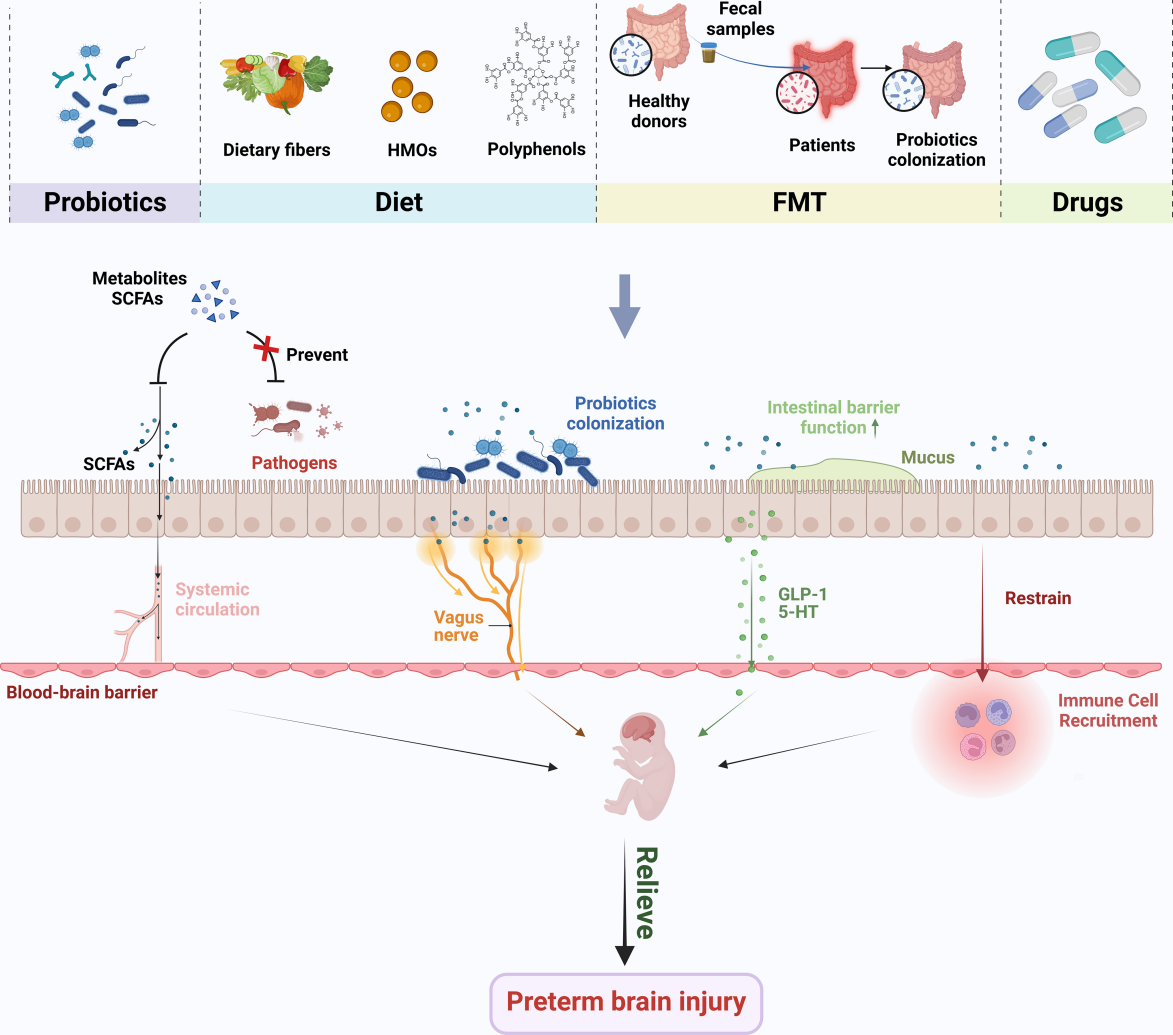
Early intervention of gut microbiota is a potential therapeutic strategy for preterm brain injury. Early microbial interventions, including probiotics, dietary supplements, FMT, and drug application, can alleviate preterm brain injury by regulating the composition of GM and intestinal environment, increasing beneficial bacterial metabolites such as SCFAs and intestinal barrier function, and regulating nerve, immune, endocrine, and other gut–brain axis pathways. *HMOs* human milk oligosaccharides; *FMT* fecal microbiota transplantation; *SCFAs* short‐chain fatty acids; *GLP‐1* glucagon‐like peptide‐1; *5‐HT* 5‐hydroxytryptamine; *GM* gut microbiota (Figure created with Biorender.com).

The early postnatal period is critical for delicate neurodevelopment and an important opportunity for therapeutic interventions for gut microbial‐associated psychiatric disorders. Previous understanding about the role of GM allows for new nutritional treatment strategies for preterm infants with brain injury. Various gut‐related interventions have already been applied to alleviate brain injury in preterm infants, including microecological agents (probiotics, prebiotics, and synbiotics), dietary interventions, FMT, and natural drugs (Figure 2). Moreover, human studies have found that adding probiotics, prebiotics, and synbiotics to dietary nutrition supports the balance of GM and improves cognition, mood, behavior, and psychology in children.^85^

### 5.1 Microecological agents

**FIGURE 3:**
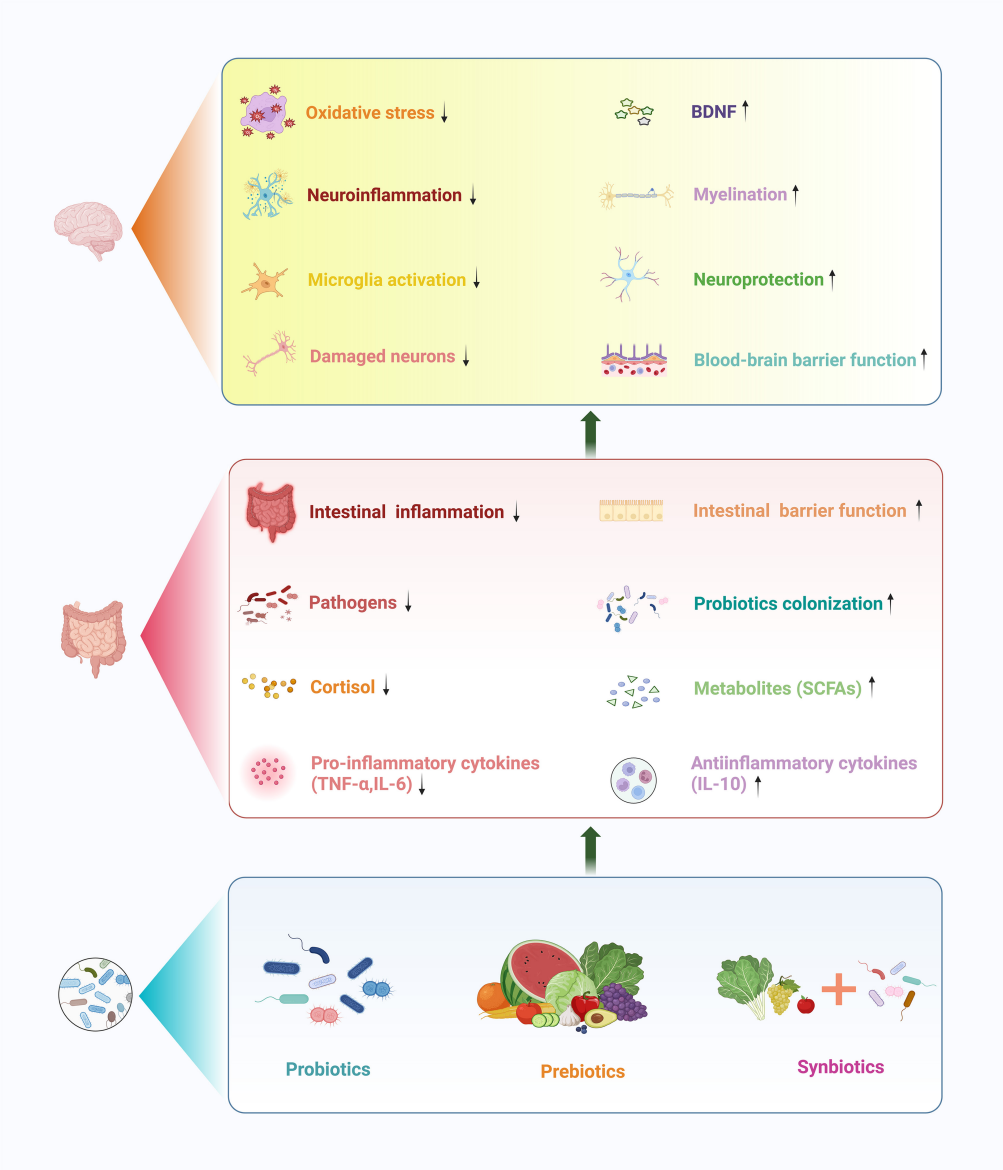
Potential mechanism of Microecological agents in preterm brain injury. Microecological agents such as probiotics, prebiotics, and synbiotics can inhibit pathogens, reduce the levels of pro‐inflammatory factors and cortisol, promote the colonization of beneficial bacteria, and increase the levels of anti‐inflammatory factors and SCFAs. Generally speaking, they play a role in anti‐inflammatory, enhancing epithelial barrier function, and diminishing gastrointestinal dysfunction. They can also intervene in the CNS development process through the gut‐microbiota‐metabolite‐brain axis pathways, including increasing BDNF levels and blood–brain barrier function, reducing neuroinflammation and exerting neuroprotective effects, thereby alleviating preterm brain injury. *BDNF* brain‐derived neurotrophic factor; *SCFAs* short‐chain fatty acids; *TNF‐α* tumor necrosis factor‐alpha; *IL‐6* interleukin‐6; *IL‐10* interleukin‐10; *CNS* central nervous system (Figure created with Biorender.com).

With the progressive research on MGBA, the application of microecological agents in neurological disorders has gained notable interest in clinical practice (Table 1). The following sections focus on the progress and challenges of using microecological agents in infants and premature infants with neurocognitive disorders (Figure 3).

**TABLE 1:** Effects of microecological agents on mental status of preterm infants.

Microecological agents	Impact on microbiota	Effects	References
Probiotics			
Bacteroidota and Lachnospiraceae	Change the integrity of the intestinal barrier and systemic metabolites	Improve cognitive‐behavioral function	[26]
Lactobacilli	Strengthen the integrity of intestinal barrier, regulate the structure and function of GM, and increase the expression of SCFAs and BDNF	Relieve chronic social defeat stress and improve cognition	[92, 96, 100]
Bifidobacteria	Improve SCFAs abundance and 5‐HT levels, reduce antibiotic resistance genes in GM of premature infants	Improve cognitive, emotional, behavioral, psychological well‐being effects	[94, 98, 101]
Bifidobacterium longum 1714	Promote the colonization of GM	Reduce stress‐related behaviors and improve cognitive function	[99]
Prebiotics			
GOS	Enrich Lactobacillus, and increase fecal butyrate and propionate levels	Relieve stress‐related behaviors	[109, 110]
FOS	Increase Probiotics colonization, and reduce endotoxemia‐induced systemic inflammation	Modulate the expression of tight junction proteins, and improve BBB integrity	[111]
Synbiotics			
Prebiotic XOS and probiotic L. paracasei HII01	Alter GM composition, attenuate gut inflammation, and increase the levels of SCFAs	Improve hippocampal plasticity and brain function, and restore cognitive function	[115]

Abbreviations: 5‐HT, 5‐hydroxytryptamine; BBB, blood–brain barrier; BDNF, brain‐derived neurotrophic factor; FOS, oligofructose; GM, gut microbiota; GOS, galactooligosaccharide; SCFAs, short‐chain fatty acids.

#### 5.1.1 Probiotics

Probiotic therapy administers to the subject active microorganisms that benefit the host's well‐being, and its effects are highly strain‐specific.^86^ Prophylactic probiotics usage is recommended to support the development of healthy GM, suppress the colonization of potentially pathogenic bacteria, and inhibit opportunistic pathogen overgrowth without altering the overall longitudinal bacterial profile in the neonatal period.^87^, ^88^ Recent studies suggest that the early microbial colonization affects brain development in preterm infants and microbiome optimization improves neurodevelopmental outcomes.^89^, ^90^ This anticipates probiotic interventions for improving neurodevelopment, although their safety remains controversial.

Probiotics is beneficial to neurodevelopment, emotion, and cognition in mice. Maternal dysbiosis in mice that are exposed to commonly used broad‐spectrum oral antibiotics (ampicillin) can affect the neurobehavioral outcomes of their offsprings, resulting in reduced social motivation and social interaction.^91^ And in maternal mice, probiotics may stimulate the expression of intestinal butyric acid, brain lactate, and BDNF in the offspring, benefiting their neurological development.^92^ Although the evidence suggests that probiotics significantly affect gut–brain axis‐mediated signaling pathways, no definitive key molecules of action have been identified up to now.

A study on structural alterations in the GM induced by antibiotic exposure in preterm infants found that early microbiome alterations would likely affect the neurological outcomes associated with premature birth.^93^ Administering complex probiotics during hospitalization reduced the diversity of antibiotic resistance genes in the GM of preterm infants and effectively prevented chronic retention of these resistance genes.^94^ However, due to the weak intestinal immunity defense of preterm infants, some probiotic strains may convert into opportunistic pathogens, increasing the risk of antibiotic resistance and leading to infections.^95^

*Lactobacillus* and *Bifidobacterium* can accelerate the maturation of gut microbial composition and immune system in extremely preterm infants. Early colonization by *Bifidobacterium* and *L. plantarum* rebalances GM, increases glutamine, γ‐aminobutyric acid, restores anxiety‐related BDNF and 5‐HT levels,^58^, ^96^ and significantly inhibits microglial activation and neuronal apoptosis, contributing to anxiolytic homeostasis.^97^ Mice experiments showed that supplementation with probiotics such as *B. shortum* strains and *L. plantarum* restored cognitive deficits, anxiety, and other abnormal social behaviors early in life.^98^ In a randomized double‐anonymized controlled trial including 40 healthy human participants, intervention with *B. longum* altered resting neural activity, which is characterized by enhanced vigor, reduced mental fatigue following social stress, providing a neural mechanism for alleviating stress responses.^99^

The premature infants are at the risk of dysbiosis and neurodevelopmental defects, for which probiotic supplementation represents a promising therapeutic strategy.^100^ A randomized intervention trial including 57 extremely preterm infants found that probiotics restored their intestinal microecology, promoted gut microbiome maturation to the level of full‐term infants, and suppressed inflammatory responses.^101^ However, limited publications in this area make it difficult to determine whether probiotics can directly improve neurodevelopmental outcomes in preterm infants, necessitating further research. Moreover, screening and isolating novel probiotics through next‐generation sequencing and bioinformatics platforms are needed for the targeted treatment of specific diseases.^102^

#### 5.1.2 Prebiotics

Prebiotics are substrates that can stimulate certain bacterial taxa or bacterial activities in the GM and can be selectively utilized and converted into beneficial substances for the host's health. They can also modulate inflammatory responses by altering the GM composition and activity, affecting mood and some psychiatric disorders. Therefore, prebiotics use is considered a practical strategy for treating neurological disorders.^103^

Interventions using prebiotics have potential advantages over using single or multiple strains of probiotics. The ingredients of prebiotics are often very stable, and they can withstand high temperature and acidic environment, which are common in the production of nutritious food and functional food.^104^, ^105^ Prebiotic ingredients typically have a longer shelf life than probiotic ingredients, and this stability also provides convenience for storage and processing.^106^ Therefore, prebiotics are gaining popularity in the field of digestive health or parenteral health.

In mice, early prebiotic intervention reduced brain lesions by upregulating the neuroprotective phenotype of microglia via the inhibition of relevant pro‐inflammatory and neurotoxic signaling pathways.^107^ A RCT of 161 infants revealed that the prebiotic intervention group cried for significantly shorter durations while awake and the 24‐h sleep pattern monitoring showed longer latency to first and second naps than in control group, respectively. Moreover, GM composition was significantly different between the two groups. These results suggest that prebiotics may influence neurodevelopment through the gut–brain axis, affecting sleep performance.^108^

So far, prebiotic supplementation mainly refers to the addition of short‐chain galacto‐oligosaccharides and long‐chain fructo‐oligosaccharides, which are metabolized by *Bifidobacteria*. Animal experiments revealed that prebiotics (galactooligosaccharide) can influence *Lactobacillus* and *Bifidobacterium* composition and induce metabolic, neurochemical, and behavioral changes in adult rodents. Continuous prebiotic supplementation may benefit the ecology and genetic stability of the GM.^109^ B‐Galactooligosaccharide supplementation in maternal mice increased exploratory behaviors and decreased hippocampal glutamate receptor gene expression in their weaning offsprings, suggesting beneficial effects on brain gene expression and behaviors in the offsprings.^110^ Another mouse model experiment showed that prebiotic (oligofructose) intake increased *Bifidobacteria* levels and decreased corticosterone release, thereby increasing the IL‐10 level, relieving brain inflammation, and maintaining BBB integrity in mice hypothalamus.^111^

Prebiotics also include fibers such as inulin and resistant starch, which are not absorbed in the small intestine but are selectively fermented by beneficial microorganisms. In the study of chronic unpredictable mild stress mice, inulin intake can protect the integrity of intestinal barrier, increase the formation of SCFAs, inhibit the decrease of BBB permeability, and regulate TLR4/MyD88/NF‐κB signal transduction to alleviate neuroinflammatory response.^112^ Therefore, prebiotic interventions might ameliorate neurological disorders in infants and preterm infants. However, with the limited data, further research is required to elucidate their exact mechanisms in neurodevelopment.

#### 5.1.3 Synbiotics

Synbiotics are food ingredients or dietary supplements that comprise probiotics and prebiotics. Synbiotics are more effective than probiotics or prebiotics alone in improving CNS‐related symptoms.^113^, ^114^ A study reported that the combination of prebiotic XOS + probiotic *Lactobacillus paracasei HII01* as a synbiotic attenuated dysbiosis and improved cognitive function by reducing intestinal inflammation, restoring hippocampal synaptic plasticity, attenuating brain mitochondrial dysfunction, and alleviating hippocampal oxidative stress and apoptosis.^115^ Given the limited clinical data and relevant clinical studies available, more preclinical and clinical evidence is needed to clarify the effects of synbiotics.

### 5.2 Dietary interventions

**FIGURE 4:**
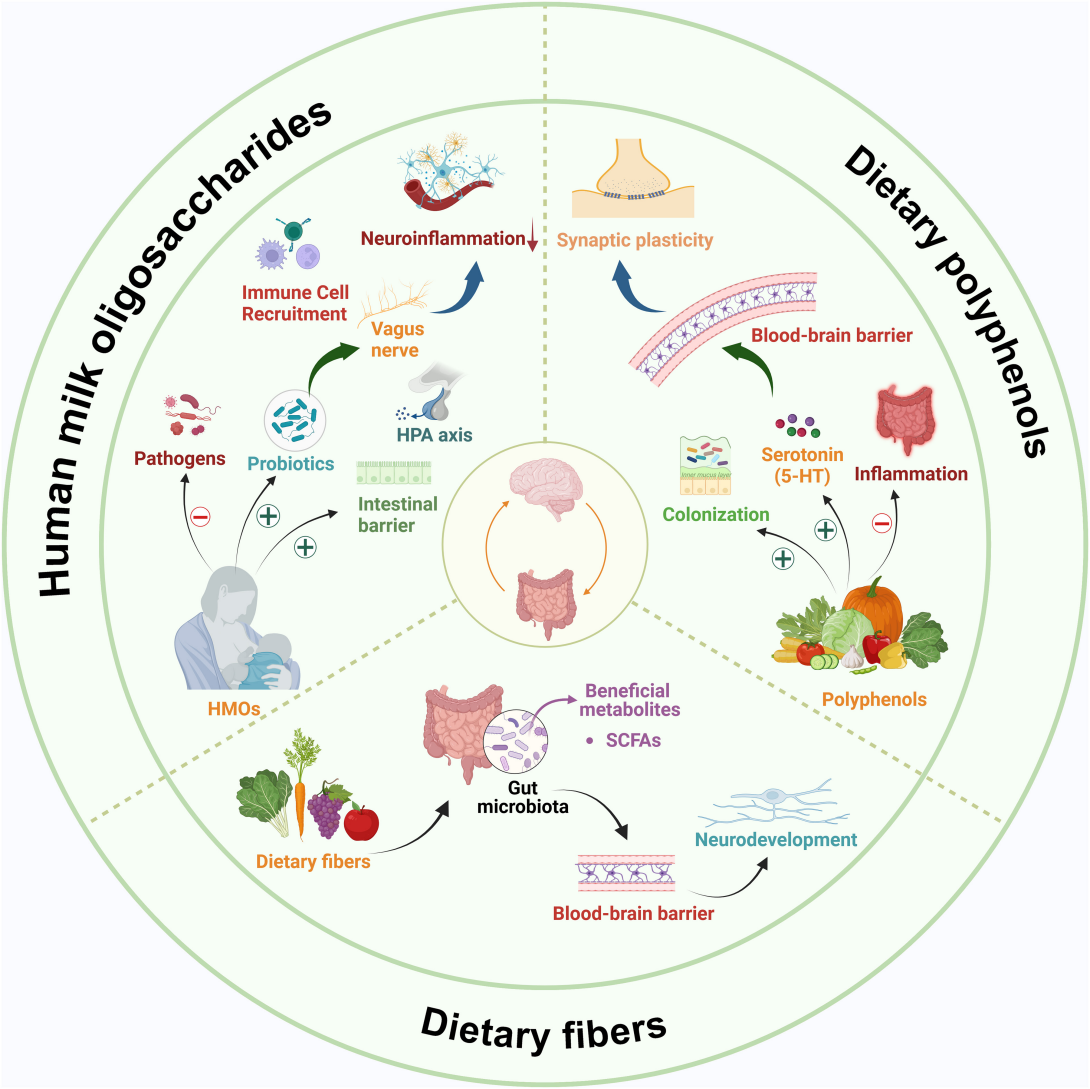
Potential mechanism of dietary intervention in preterm brain injury. The potential mechanism of dietary intervention in brain injury in premature infants. Dietary fiber, polyphenols, and HMOs can affect the composition and diversity of gut microbiota, and increase the levels of beneficial bacteria and their metabolites (SCFAs). These metabolites can affect the vagus nerve pathway, immune signaling pathway, HPA axis, and blood–brain barrier function, thereby alleviating neuroinflammation, enhancing synaptic plasticity and neurodevelopment. *HMOs* human milk oligosaccharides; *HPA* hypothalamic–pituitary–adrenal; *SCFAs* short‐chain fatty acids; *5‐HT* 5‐hydroxytryptamine (Figure created with Biorender.com).

A symbiotic relationship between the GM and the host starts early in life, with initial perturbations affecting neurodevelopment. Dietary interventions are involved in brain development and function through microbial‐dependent and independent mechanisms (Table 2). The emerging role of dietary interventions as a non‐invasive and practical adjunct treatment for neurocognitive disorders has been highlighted in early psychiatric disorders. They are usually administered as nutritional supplements, including human milk oligosaccharides (HMOs), dietary fiber, and dietary polyphenols^116^ (Figure 4).

**TABLE 2:** Effects of dietary intervention and FMT on mental status of preterm infants.

Dietary intervention and FMT	Impact on microbiota	Effects	References
Dietary intervention			
HMOs	Promote the colonization of beneficial bacteria, and improve the maturation of the intestinal epithelial barrier	Improve cognitive development	[118, 119, 120, 121]
Dietary fibers	Regulate the GM diversity, facilitate recovery of SCFAs‐producing bacteria, and promote BBB integrity	Improve the behavioral deficits, synaptic damage, and cognitive performance	[125, 126]
Dietary polyphenols	Enrich beneficial bacteria, upregulate the expression of SCFAs	Restore the integrity of the BBB, and boost mental and brain function	[131, 132, 133]
FMT	Reshape GM, enhance intestinal barrier integrity, upregulate expression of fecal SCFAs	Improve neurological functions and neuronal axonal regeneration	[138]

Abbreviations: BBB, blood–brain barrier; FMT, fecal microbiota transplantation; GM, gut microbiota; SCFAs, short‐chain fatty acids.

#### 5.2.1 Human milk oligosaccharides

HMOs are ideal supplements because of their good stability and ability to withstand gastric acid and high temperatures in pasteurization. HMOs have been reported to optimize *Bifidobacteriaceae* and *Bacteroidaceae* colonization, suppress pathogen invasion, promote immune development, and regulate microbiota‐gut‐brain communication.^117^, ^118^ In a pig model fed with HMOs, HMO‐diet‐induced intestinal alterations were associated with changes in gene expression encoding the BBB, endocrine function, and SCFA receptors in brain tissue.^119^ Another pig study showed that gut microbes could leverage metabolites produced by HMOs to induce structural changes in the brain and enhance cognitive function.^120^ Additionally, human studies have demonstrated multiple roles for HMOs as modulators of the GM in children, influencers of colonization of *Bifidobacteria* in the infant gut, and facilitators of neonatal neurological development.^121^, ^122^

#### 5.2.2 Dietary fiber

Dietary fiber can be used as an additive in formula which can be fermented by GM into host‐absorbable SCFAs in the colon.^123^ As key signaling molecules for gut–brain communication, SCFAs readily cross the BBB to stimulate BDNF expression, thereby regulating the development, and maturation of neurons and microglia and modulating major neurodevelopmental processes during gestation.^124^ Mice experiments found that high dietary fiber intake improved cognitive and social behavioral deficits in the offspring of obese maternal mice by modulating the composition of the offspring's *Clostridiales* and *Bacteroidales* and SCFAs.^125^ Moreover, sodium butyrate promoted the recovery of SCFA‐producing bacteria in neonatal rats with hypoxic–ischemic brain damage, facilitating the recovery of cerebral cortex and hippocampus.^126^ However, not all fibers are beneficial; unfermented fibers may elicit the production of pro‐inflammatory factors by immune cells, activating the NLRP3 and TLR2 pathways and exacerbating inflammation in Inflammatory bowel disease (IBD).^127^ Therefore, although modulating GM through dietary fiber supplementation is possible, the diverse findings of relevant studies are insufficient to back up individualized regimen.

#### 5.2.3 Dietary polyphenols

Dietary polyphenols, primarily from plant sources, can inhibit pro‐inflammatory cell signaling pathways and regulate GM. They are also recognized as exogenous molecules capable of modulating neurogenesis. Active metabolites in the brain that come from polyphenol biotransformation are processed by GM. Polyphenol metabolites can either directly cross the BBB or indirectly modulate the cerebrovascular system, thereby improving cognitive function and relieving neuropsychiatric disorders.^128^, ^129^

A review of animal and clinical studies demonstrated that dietary polyphenols can act as drugs for the treatment of intestinal and neurodevelopment‐related diseases.^130^ Resveratrol, a natural polyphenol, is involved in downregulation of *Bacteroidetes* and *Lachnospiraceae* and the regulation of Glucagon‐like peptide‐1, and 5‐HT levels, thus restoring the integrity of the BBB, inhibits the activation of microglia, and suppresses CNS inflammation.^131^, ^132^ Polyphenols are also crucial for neurodevelopment in uterus and during lactation.^133^ They can induce adult hippocampal neurogenesis and enhance learning and memory by increasing synaptic plasticity and promoting long‐term hippocampal enhancement, while hippocampal neurogenesis in the offsprings is affected by perinatal maternal nutrition.^134^ In conclusion, the supplementation of plant‐active substance polyphenols provides a novel perspective for the treatment of early‐life brain injury.

### 5.3 Fecal microbiota transplantation

**FIGURE 5:**
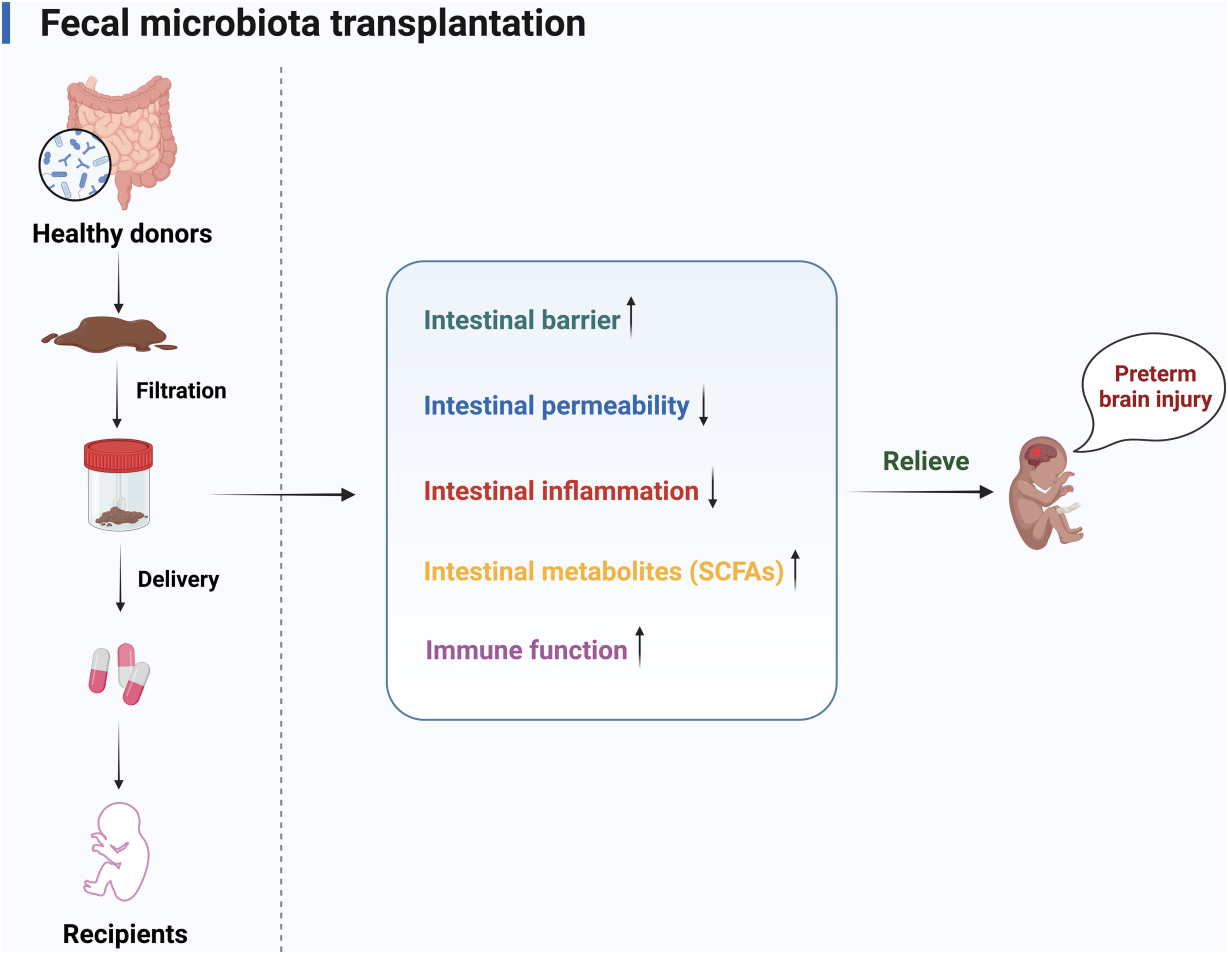
Potential mechanism of FMT in preterm brain injury. FMT involves the transfer of fecal bacteria from healthy individuals to individuals with pathological conditions, which can reduce intestinal inflammation and permeability, increase intestinal beneficial metabolites and barrier function to relieve neurological diseases. It has been found to be an effective method to reduce the pathophysiology of preterm brain injury. *FMT* fecal microbiota transplantation; *SCFAs* short‐chain fatty acids (Figure created with Biorender.com).

FMT is the delivery of various intestinal microorganisms, metabolites, and natural antimicrobial agents isolated from the feces of healthy individuals (donors) into the intestine of patients (recipients) by oral administration, enema, and colonoscopy. FMT helps re‐establish the balance of GM, repair the intestinal mucosal barrier, control inflammatory responses, and regulate host immunity^135^ (Figure 5). Numerous studies have confirmed the effectiveness and safety of FMT for treating recurrent *Clostridium difficile* infections,^136^ IBDs,^137^ and other gastrointestinal diseases. FMT can modulate neurological disorders driven by dysbiosis, and it may enhance neural regeneration in mice with spinal cord injury by restraining blood‐spinal cord barrier damage, upregulating neurotrophic factors, alleviating spinal cord ischemia, and promoting neuronal survival^138^, ^139^ (Table 2).

FMT is a treatment option for neonatal necrotizing enterocolitis (NEC) and related mental disorders in preterm infants based on its regulatory role on GM. NEC is characterized by dysbiosis (i.e., increased TLR4 activity) and is associated with a significant risk of neurodevelopmental disorders.^140^ In a rat model of NEC, FMT before or after induction of NEC significantly alleviated inflammation, oxidative stress, and intestinal mucosal damage, suggesting that FMT may be a potential therapy for NEC.^141^ Experimental FMT in neonatal preterm piglets reduced NEC risk, but the effect was donor‐specific.^142^ This process may be related to the promotive effect of early FMT intervention on the development of the innate and adaptive immune systems and the maturation of the GM in piglets.^143^ Although FMT has been widely used in clinical practice, transplanting live bacteria increases infection risk, making it imperative to develop donor screening protocols to ensure the safety of this therapy.^144^ Although there is no direct evidence supporting FMT in NEC‐related brain injury, future studies are expected to advance and optimize the application of FMT in preterm brain injury, aiming at reducing long‐term neurological complications.

### 5.4 Natural drugs

There are intricate interactions between drugs and GM, with the gut–brain axis being a possible route for conveying the benefits of drugs for brain health (Table 3). Plant‐derived anthocyanins may prevent cognitive deficit‐related behaviors by correcting dysbiosis, restoring the intestinal ecological and functional microenvironment, and inhibiting inflammation mediated by immune cells.^145^ Apart from alleviating cognitive impairment, plant root‐derived oxymatrine may alleviate clinical symptoms and pathological manifestations of multiple sclerosis by correcting microbiota dysbiosis, suppressing immune cell‐mediated inflammation, and remodeling the gut–brain axis.^146^ Another natural plant antitoxin, pterostilbene, could reduce the release of inflammatory factors by decreasing lipopolysaccharide levels and modulating TLR4/NF‐κB signaling pathways in the colon and brain, thereby inhibiting oxidative stress damage, astrocyte and microglia activation, and dopaminergic neuron loss.^147^ Natural plant‐derived active agents are promising in treating neurological disorders through the modulation of GM, providing theoretical basis for its future therapeutic value.

**TABLE 3:** Effects of drugs on mental status of preterm infants.

Drugs	Impact on microbiota	Effects	References
Oxymatrine	Remodel GM homeostasis	Decrease BBB permeability, and ameliorate experimental autoimmune encephalomyelitis	[146]
Pterostilbene	Increase the levels of SCFAs and maintain normal intestinal permeability	Ameliorate cognitive dysfunction, and improve both memory and learning ability	[147]
Dimethyl itaconate	Reshape the GM profile and maintain gut immune homeostasis	Optimize synapse and cognition‐associated genes, and improve cognitive impairment	[148]
Dectin‐1 ligand pachyman	Regulate gut immunity homeostasis and control Treg cell differentiation in the intestine	Prevent stress‐induced behavioral abnormalities	[149]
Diosgenin	Upregulate the relative abundance of Lactobacillus and the expression of SCFAs, and restore the activity of the HPA axis	Alleviate inflammation‐related brain injury behavior	[150]

Abbreviations: BBB, blood–brain barrier; GM, gut microbiota; HPA, hypothalamic–pituitary–adrenal; SCFAs, short‐chain fatty acids.

Microbiota–gut–brain axis links multiple biofeedback systems through which many drugs work on CNS disorders. For example, dimethyl itaconic acid can significantly reduce macrophage infiltration and expression of pro‐inflammatory cytokines (TNF‐α, IL‐1β, and IL‐6) in high‐fat‐diet mice, thereby increasing propionate and butyrate levels to restore the ecological and functional microenvironment of the intestine and improve cognition.^148^ However, currently no neuroprotective strategies have been validated to prevent inflammation‐related brain damage in preterm infants. Natural drugs that support brain health may provide essential benefits. A decrease in certain *Lactobacilli* associated with increased differentiation and accumulation of IL‐17‐producing γδ17 T cells in the meninges was observed in a study analyzing the microbiome characteristics of stress‐sensitive mice and patients with major depression. This study demonstrates that this alteration promoted stress‐induced social avoidance behaviors in mice and oral administration of the dectin‐1 ligand porin glucan modulated the stress response to chronic social stress.^149^ Another study reported that plant‐derived diosgenin might reduce inflammation‐related brain injury by regulating the *Firmicutes* and *Lactobacillus*, enhancing neurotrophic functions, and suppressing inflammatory and neuroendocrine activities.^150^

## 6 CONCLUDING REMARKS AND FUTURE PERSPECTIVE

In conclusion, the GM plays a crucial role in the pathogenesis of central nervous system diseases and might be considered as a new organ, namely the second brain. The related changes in the composition of gut microbiota and their metabolites may affect neurodevelopment by activating the vagus nerve, stimulating intestinal endocrine cells and immune signals, as well as the function of the blood–brain barrier. However, due to the complexity of microbiota composition and their activities, the mechanisms are far from clearly explored. As different neurodevelopmental disorders may reflect different microbial patterns associated with specific neurological conditions, targeted microbial intervention approaches based on the hypothesis of appropriate recipient‐donor matching of gut microbial profiles could be explored concerning the neurology of preterm infants. New analyzing and sequencing methods may be of some help. Future neurotherapeutic research will provide more important information about the GM and neurological diseases in preterm infants. The application of microbial interventions, such as FMT, probiotics, and prebiotics as a promising treatment strategy, also presents some concerns and challenges. Therefore, the safety and tolerance of microbial interventions should be carefully evaluated to avoid adverse effects in the future and to promote the success of targeted microbial therapy clinical trials in preterm brain injury.

## AUTHOR CONTRIBUTIONS

LL collected the literature, conceptualized the title, prepared the initial draft, designed the figures, and revised the manuscript according to YYS's comments, modifications, and suggestions. TJL proofread the language problems. All authors read and approved the final manuscript.

## FUNDING INFORMATION

This work was supported by the National Natural Science Foundation of China (82171709 and 81801500), the 345 Talent Project of Shengjing Hospital (M1392 and M1415), Key R&D Guidance Plan Project in Liaoning Province (2020JH1/10300001), and Natural Science Foundation of Liaoning Province (No. 2022‐MS‐207).

## CONFLICT OF INTEREST STATEMENT

The authors declare that they have no competing interests.

## Data Availability

Data sharing is not applicable to this article as no new data were created or analyzed in this study.
